# Synaptic and functional alterations in the development of mutant huntingtin expressing hiPSC‐derived neurons

**DOI:** 10.3389/fmolb.2022.916019

**Published:** 2022-07-19

**Authors:** Margarita C. Dinamarca, Laura Colombo, Natalia E. Tousiaki, Matthias Müller, Eline Pecho-Vrieseling

**Affiliations:** ^1^ Department of Biomedicine, University of Basel, Basel, Switzerland; ^2^ Novartis Institute for Biomedical Research, Basel, Switzerland

**Keywords:** neurodevelopment, huntington (disease), human iPS cell, synapsis formation, neurodegeneration, mutant huntingtin

## Abstract

Huntington’s disease (HD) is a monogenic disease that results in a combination of motor, psychiatric, and cognitive symptoms. It is caused by a CAG trinucleotide repeat expansion in the exon 1 of the huntingtin (*HTT*) gene, which results in the production of a mutant HTT protein (mHTT) with an extended polyglutamine tract (PolyQ). Severe motor symptoms are a hallmark of HD and typically appear during middle age; however, mild cognitive and personality changes often occur already during early adolescence. Wild-type HTT is a regulator of synaptic functions and plays a role in axon guidance, neurotransmitter release, and synaptic vesicle trafficking. These functions are important for proper synapse assembly during neuronal network formation. In the present study, we assessed the effect of mHTT exon1 isoform on the synaptic and functional maturation of human induced pluripotent stem cell (hiPSC)-derived neurons. We used a relatively fast-maturing hiPSC line carrying a doxycycline-inducible pro-neuronal transcription factor, (iNGN2), and generated a double transgenic line by introducing only the exon 1 of *HTT,* which carries the mutant CAG (*mHTTEx1*). The characterization of our cell lines revealed that the presence of mHTTEx1 in hiPSC-derived neurons alters the synaptic protein appearance, decreases synaptic contacts, and causes a delay in the development of a mature neuronal activity pattern, recapitulating some of the developmental alterations observed in HD models, nonetheless in a shorted time window. Our data support the notion that HD has a neurodevelopmental component and is not solely a degenerative disease.

## Introduction

Huntington’s disease (HD) is an autosomal dominant inherited, neurodegenerative disease that causes a progressive accumulation of motor, cognitive, and psychiatric symptoms. It develops with a 100% penetrance when the number of *CAG* triplets in exon 1 of the *HTT* gene exceeds 35 repeats. This repeat is translated into a pathogenic polyglutamine stretch in the N-terminal of the huntingtin (HTT) protein, which causes it to misfold and aggregate (Mac Donald et al., 1993). HD belongs to the family of protein misfolding diseases (PMDs) together with polyglutamine diseases, amyotrophic lateral sclerosis, Alzheimer’s-, and Parkinson’s disease. PMDs share a delayed onset in mid-adulthood or later despite the expression, at least in hereditary cases, of the disease-driving protein from the first days of life. This raises the question of whether early events might set the stage for later disease, in particular, because wild-type HTT is essential for development, at least in mice (Duyao et al., 1995; Zeitlin et al., 1995; Reiner et al., 2003). Indeed, a growing body of evidence supports a developmental component in HD. Mutant HTT (mHTT) impairs neuronal progenitor cell division, neuronal migration, and synapse development (Godin et al., 2010; McKinstry et al., 2014; Barnat et al., 2017). The expression of mHTT solely during early life is sufficient to produce HD features in adult mice (Arteaga-Bracho et al., 2016; Molero et al., 2016). Importantly, human fetuses that carried the HD mutation have revealed developmental abnormalities of the cortex (Barnat et al., 2020).

Studies in neurons derived from HD human induced pluripotent stem cells (hiPSCs) have identified changes in gene expression that support an altered developmental program (Ring et al., 2015; Consortium, 2017). Early telencephalic induction and late neural identity are affected in cortical and striatal populations in HD-derived induced pluripotent stem cell lines. In addition, gene expression analysis in organoids showed that HD organoids were correlated with an immature ventricular zone/subventricular zone, whereas control organoids developed mature human fetal cortical areas (Conforti et al., 2018).

The localization of HTT at the synapse, its prominent role in both pre- and postsynaptic function, and the finding that synaptic dysfunction occurs before neuronal atrophy and behavioral symptoms in HD (Di Figlia et al., 1995; Sun et al., 2001; Marcora and Kennedy, 2010; Rozas et al., 2011; Barron et al., 2021), led us to investigate whether mHTT affects early developmental properties of synaptogenesis in human neurons. We used a hiPSC line carrying a doxycycline-inducible pro-neuronal transcription factor (*iNGN2*) stable transfected with *mHTT exon 1* (*mHTTEx1*). The *iNGN2* line generates neurons with synaptic potentials already on day *in vitro* (DIV) 14 (Nehme et al., 2018). The pathogenic *CAG* repeat length in exon 1 of *HTT* causes incomplete splicing of the mRNA, which results in a highly toxic mHTTEx1 isoform (Neueder et al., 2018). The expression of only exon 1 with a pathogenic *CAG* repeat is sufficient to induce numerous HD-related phenotypes in many species (Wang et al., 2006; Barbaro et al., 2015; Neueder et al., 2018). We analyzed the neuronal maturation and early development of both synaptic markers and intrinsic functional properties and found that mHTTEx1-expressing neurons had a significantly altered morphological and molecular phenotype at both pre- and postsynaptic sides. Furthermore, they formed fewer synaptic contacts and showed a delay in the development of a mature current-induced action potential firing pattern. This work suggests that the presence of mHTTEx1 impairs neuronal development in human neurons.

## Material and methods

### iPSC culture and characterization

hiPSCs were previously generated from healthy adult human dermal fibroblast lines from a 32-year-old female from Invitrogen (C-013-5C). hiPSCs were maintained on Matrigel (354277, Corning)-coated dishes with mTeSR 1 medium (05851, STEMCELLS Technologies) supplemented with Pen/Strep 1% (15070-063, Thermo Fisher).

### Generation and differentiation of neurons

hiPS (passage 21–25) cells were plated on Matrigel in a proliferation medium comprising DMEM/F12 with Glutamax (10565-018, Gibco) supplemented with 2% B27 (17504–044, Thermo Fisher) and 1% N2 (17502-048, Thermo Fisher), 1% Pen/Strep (15070-063, Thermo Fisher) supplemented with 10 ng/ml hEGF (PHG0315, Thermo Fisher), 10 ng/ml hFGF (CTP0263, Invitrogen), with 10 µM Rock inhibitor (RI) for 1  day and 1 μg/ml doxycycline for 3  days, then progenitors were kept frozen in Cryostor freezing medium (07930, STEMCELL technology) or plated for immediate experiments as follows: 3.0 ×  10^5^ iND3 are plated in 24-well plate format with a neuronal differentiation medium comprising Neurobasal Medium (21103049, Thermo Fisher) + B27 with Vit. A (17504-044, Invitrogen) + N2 supplements (17502-048, Invitrogen) + Pen/Strep/Glutamax 1% supplemented with BDNF and GDNF (all from R&D at 10 ng/ml). Starting from day 2 of co-culture, the medium was changed every other day. An experiment was considered an “independent experiment” when a full differentiation process was performed from an independent hiPSC vial.

### Plasmid generation

The human Huntingtin exon 1 carrying pathological 72 glutamines is fused to mCherry (HTTEx1Q72-mCherry) under the *CAG* promoter in a PiggyBac (PB) plasmid. The plasmid was obtained by gene synthesis and cloned into the PB backbone by Life Technology Europe BV. The PB plasmid was nucleofected in hiPS *NGN2*.

### Generation HTTEx1Q72-mCherry stable lines

A single-cell suspension of hiPS is collected upon TrypLE Express Enzyme (12604-013, Gibco) detachment (5′ at 37°C). A total of 1  ×  10^6^ cells were resuspended in 100 μl of the nucleofection hESC solution 1 (Human Stem Cell Nucleofector^®^ Kit 1/Lonza #VPH-5012) where 5 μg of plasmids were added previously: 4 μg PB construct 1 μg Dual helper (expressing transposase). Nucleofection was performed using program B-016 on the Amaxa nucleofector II. Cells were immediately seeded after transfection into 6 cm Matrigel-coated dishes containing mTESR1 medium supplemented with 10 μM RI. Puromycin selection (1 μg/ml) was started 48–72 h later. Clones were picked after 10 days. The clone was seeded in a new Matrigel-coated35-mm dish to amplify the new stable lines. The presence of HTTEx1Q72-mCherry was checked via Western blotting using the Mab5492 antibody.

### Electrophysiology

The whole-cell patch-clamp technique was used to record current-induced action potentials of neurons on DIV 7, 14, and 21. Cultures were taken from the incubator and transferred to the recording chamber with artificial cerebral spinal fluid (ACSF) containing (in mM) 125 NaCl, 25 NaHCO_3_, 2.5 KCl, 1.25 NaH_2_PO_4_, 2 MgCl_2_, 2.5 CaCl_2,_ and 11 glucose, pH 7.4, constantly bubbled with 95% O_2_ and 5% CO_2_; 315–320 mOsm. The cells were maintained at 30–32 °C and allowed to adapt for 20 min before recordings. Neurons were visualized with an LNScope (Luigs & Neumann) equipped with an oblique illumination condenser, a 60× objective (LUMPplanFI, NA 0.9), and a reflected illuminator (Olympus). Patch electrodes (5–7 MΩ) were pulled from borosilicate glass tubing and filled with an intracellular solution containing (in mM) 125 K-gluconate, 20 KCl, 10 HEPES, 10 EGTA, 2 MgCl_2_, 2 Na_2_ATP, 1 Na_2_-phosphocreatine, and 0.3 Na_3_GTP, pH 7.2 (with KOH); 312.3 mOsm. The firing pattern of neurons was determined by holding the membrane potential at −60 mV and injecting step currents of 800 msec. Current-induced action potentials were recorded using a Multiclamp 700 B amplifier (Molecular Devices) and digitized at 10 kHz. Recordings were performed at 30–32°C in oxygenated ACSF. Igor Pro software (version 6.3, Wavemetrics) was used for both data acquisition and offline analysis. Multiple neurons from at least three coverslips and two independent differentiations were recorded. Firing patterns were categorized according to what has been described previously (Zhao and Wu, 1997; Xie et al., 2018; Dinamarca et al., 2021) (for examples see Figure 3B).

### Antibodies and dyes

For western blot: 1:2000 doublecortin (Dcx) (4,604, Cell signaling), 1:1000 NeuN (MAB377, Merck), 1:5000 β1-tubulin (T5201, Sigma), 1:5000 MAB5492 (MAB5492, Sigma-Aldrich), and 1:5000 β-actin (A5441, Sigma). For immunofluorescence: 1:2000 Bassoon (141 013, Synaptic Systems), 1:1000 neurofilament M (171 204, Synaptic systems); 1:5000 mCherry (ab205402, Abcam), 1:2000 Map2 (ab5392, Abcam), and 1:1000 (MAB2263, Merk). All the secondary antibodies were Alexa conjugated from Jackson ImmunoResearch and used 1:1000 for 1 h at RT.

### Western blot

iPSC-derived neurons were harvested at different time points, washed twice with ice-cold PBS, and subsequently lysed in RIPA buffer supplemented with a complete EDTA-free protease inhibitor mixture (11873580001, Roche). Lysates were incubated on ice for 15 min and cleared via centrifugation (10,000  ×  *g*) for 10 min at 4 °C. Supernatants were collected and the protein concentration was determined using a BCA assay kit (Thermo Scientific Pierce, 23227). Lysates were resolved using standard SDS-PAGE gels and after blocking, blots were incubated with primary antibodies overnight at 4 °C. After washing, the blots were incubated with secondary antibodies and visualized using a SuperSignal Femto chemiluminescent detection kit (Thermo Scientific) in Odyssey Infrared Imager (LiCor, 9120).

### Immunofluorescence

Cells on glass coverslips (in format 24-well plates with 3  ×  10^5^ neurons density) were fixed for 5 min in 4% PFA/4% sucrose at RT, permeabilized with PBS^+/+^ (D8662, Sigma, supplemented with 1 mM MgCl2 and 0.1 mM CaCl2)/Triton−0.1%, blocked with 5% BSA in PBS^+/+^, and labeled with primary antibodies in PBS^+/+^ (D8662, Sigma) and 5% BSA overnight at 4°C and secondary antibodies for 1 h RT. PBS^+/+^ washing was performed after each antibody incubation. Coverslips were mounted on glass slides in Prolong (P36930, Invitrogen).

### Image acquisition and analysis

Fluorescence signals were imaged with a Zeiss LSM-700 system with a Plan-Apochromat 40  ×/NA 1.30 oil DIC, using Zen 2010 software. All images were acquired with identical microscope settings within individual experiments. Saturation was avoided by using image acquisition software to monitor intensity values. For any image adjustment, identical settings were always applied to all cells, irrespective of the genotype. For quantification, values were averaged from multiple cells from at least three independent culture preparations.

Quantification of images was done using ImageJ open-source software (version 2.1.0). The images were background subtracted and after setting an automated threshold, the “Analyze Particles” plugin was used to determine the number of puncta of synaptic markers.

### Statistical analysis

Data analysis was performed with GraphPad Prism version 8.0 (GraphPad Software, La Jolla, CA). Individual datasets were tested for normality with the Shapiro–Wilk, D’Agostino–Pearson, or Kolmogorov–Smirnov test. Statistical significance of differences between groups was assessed by unpaired two-tailed Student’s t-test or two-way ANOVA mixed-effect model with the Geisser–Greenhouse correction and Sidak’s multiple comparison test, as indicated in the figure legends. Data are presented as mean ± standard error of the mean (s.e.m.).

## Results

**FIGURE 1:**
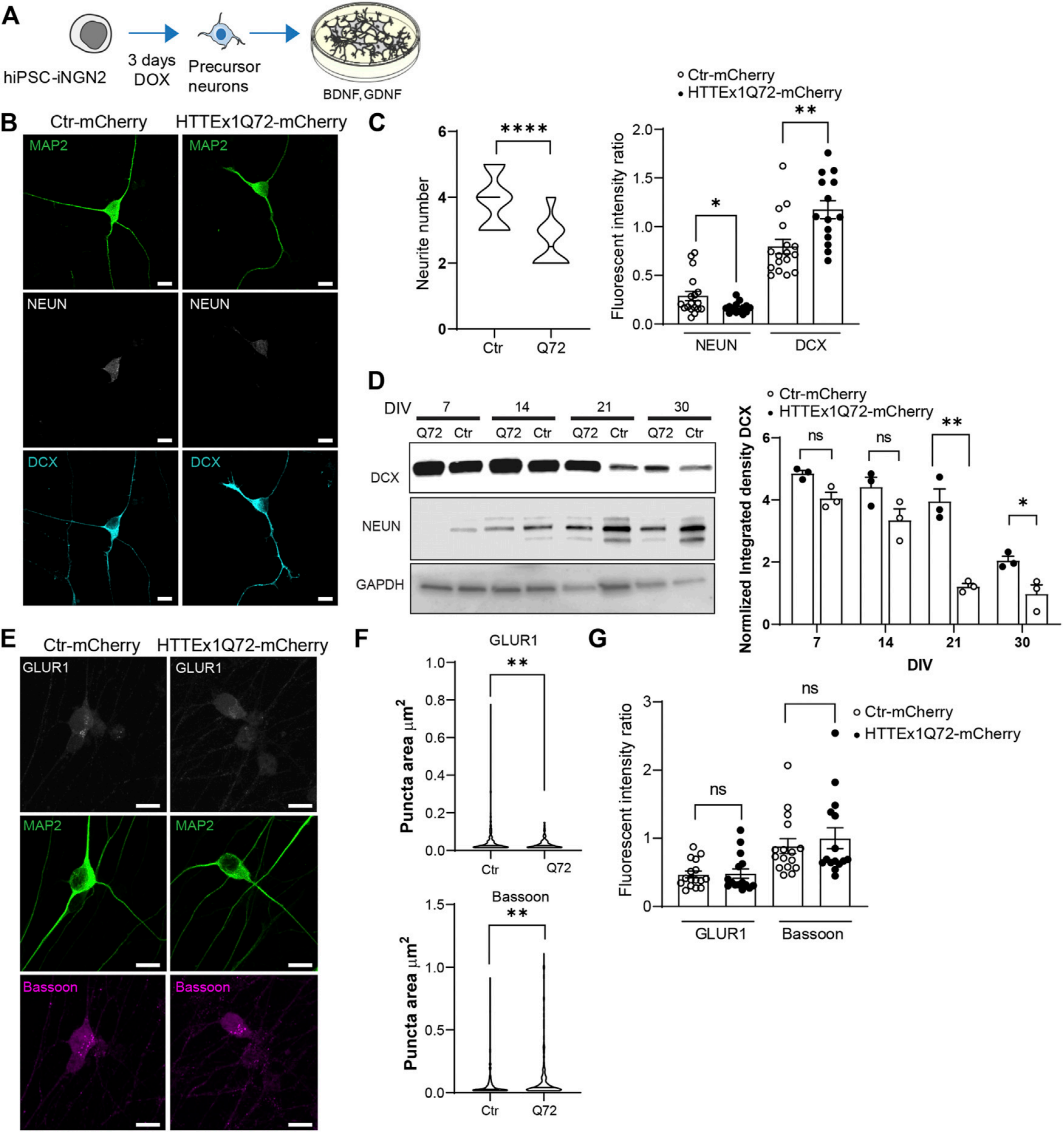
HTT Ex1Q72-mCherry neurons show a decrease in maturation compared to Ctr-mCherry neurons. **(A)** Schematic experimental approach to follow the culture of hiPSC iNGN2 cells to forebrain neurons. **(B)** Indirect immunofluorescence representative images of Ctr-mCherry (Ctr) and HTTEx1Q72-mCherry (Q72) neurons on DIV 7 visualizing MAP2, NEUN, and DCX proteins. **(C)** (left) Neurite number quantification in Ctr-mCherry and HTTEx1Q72-mCherry neurons on DIV 7 (*n* = 15 cells from three independent cultures). **(C)** (right) Fluorescence intensity ratio quantification, NEUN end DCX fluorescence intensity was normalized to MAP2 intensity, comparing Ctr-mCherry and HTTEx1Q72-mCherry neurons on DIV 7 (*n* = 15–20 cells from three independent cultures). **(D)** (left) Representative Western blot of total homogenate from Ctr-mCherry and HTTEx1Q72-mCherry neurons at indicated time points, showing the expression pattern of DCX, NEUN, and GAPDH. **(D)** (right) Quantification of normalized integrated density of DCX at different time points (n = 73). **(E)** Indirect immunofluorescence representative images of Ctr-mCherry and HTTEx1Q72-mCherry neurons on DIV 7 visualizing MAP2, presynaptic marker Bassoon and postsynaptic marker GLUR1. **(F)** Puncta area quantification of GLUR1 and Bassoon in the soma of Ctr-mCherry and HTTEx1Q72-mCherry neurons on DIV 7 (*n* = 200–400 puncta from cells from three independent cultures). **(G)** Fluorescent intensity of GLUR1 and Bassoon normalized to MAP2 intensity in Ctr-mCherry and HTTEx1Q72-mCherry neurons on DIV 7 (*n* = 15–16 cells from three independent cultures). Scale bar = 10 µm. All averaged data are shown as mean ± s.e.m. (*p < 0.05, **p < 0.005, ***p < 0.0001. ns = non-significant Student’s (Abbr: MAP2, microtubule-associated protein 2, DCX: doublecortin, GLUR1, Glutamate receptor 1 subunit).

A hallmark of HD is early synaptic dysfunction, including altered neurotransmitter release and NMDA and AMPA-receptor signaling (Milnerwood and Raymond, 2010; Huang et al., 2020; Barron et al., 2021). The fact that mHTT is present during neuronal development raises the interest to assess whether early developmental processes, important for appropriate assembly of neuronal networks, are altered in HD patients. We used an hiPSC line bearing a doxycycline-inducible pro-neuronal transcription factor, *NGN2* (*iNGN2*), and generated a double transgenic line by introducing a *HTTEx1Q72* fused to *mCherry* (*iNgn2; HTTEx1Q72-mCherry*, hereafter HTTEx1Q72-mCherry) (Nehme et al., 2018; Dinamarca et al., 2021). We analyzed three clones for their expression of HTTEx1Q72-mCherry and decided to use, in this study, clone#72, which showed an intermediate level of protein expression and has been shown to have a moderate aggregate formation over time (Supplementary Figure S1) (Dinamarca et al., 2021). As a control line, we used *iNGN2* hiPSCs expressing mCherry (Ctr-mCherry). These cell lines were differentiated into forebrain-type neurons (Figure 1A). We analyzed the protein levels of the glutamatergic and gabaergic cell type-specific markers vGLUT1 and GAD65, respectively, and the expression of the postsynaptic glutamatergic receptor marker AMPA receptor subunit GLUR1, and the pan-neuronal postsynaptic density marker PSD-95. These proteins were equally expressed between the control and mHTTEx1 cultures on DIV 7. GLUR1, PSD95, and vGLUT1 increased from DIV7 to 21 in control cultures. In HTTEx1Q72-mcherry cultures, this increase was less evident. For GAD65, the protein levels were not different over time and between both groups (Supplementary Figure S2).

To assess the maturation state of the hiPSC-derived neurons on DIV 7, we counted the neurite number and labeled the neurons for the neuronal precursor marker doublecortin (DCX) and the mature neuronal marker NEUN. Neurons expressing HTTEx1Q72-mCherry showed a significant decrease in the neurite number compared with Ctr-mCherry neurons (Figures 1B,C, left panel). In addition, we observed that HTTEx1Q72-mCherry showed a significantly higher expression of DCX than Ctr-mCherry neurons, and a reduced expression of NEUN on DIV 7 (Figures 1B,C, right panel). To assess whether the different protein expression levels result in a distinct maturation profile, we performed western blotting on DIV 7, 14, 21, and 30. From DIV 21 we found a decrease in DCX in Ctr-mCherry compared to DIV 7 and 14, while in HTTEx1Q72-mCherry, this decrease only occurred on DIV 30. Actually, on DIV 21 and 30, there is a significant increase in the expression levels of DCX in HTTEx1Q72-mCherry neurons compared to the control (Figure 1D, right panel). Ctr-mCherry neurons already showed the expression of NEUN on DIV 7, in HTTEx1Q72-mCherry, we observed a clear expression first on DIV 14 (Figure 1D). Next, we analyzed the presence of synaptic markers on DIV 7. At this time point of the culture, they are still mainly localized in the soma of the neurons and have not yet been distributed to the synaptic sites. Thus, we measured the intensity of the presynaptic scaffold protein Bassoon and the postsynaptic AMPA-receptor subunit GLUR1 in the soma. The intensity of both markers was not significantly different between Ctr-mCherry and HTTEx1Q72-mCherry neurons. However, the area of the GLUR1 puncta in HTTEx1Q72-mCherry neurons were significantly smaller than those in Ctr-mCherry neurons. On the other hand, the Bassoon puncta area was significantly bigger in HTTEx1Q72-mCherry than in Ctr-mCherry neurons. Our results suggest that hiPSC-neurons expressing mHTTEx1 Q72-mCherry have a significant decrease in maturation parameters compared to Ctr-mCherry neurons, already on DIV 7.

**FIGURE 2:**
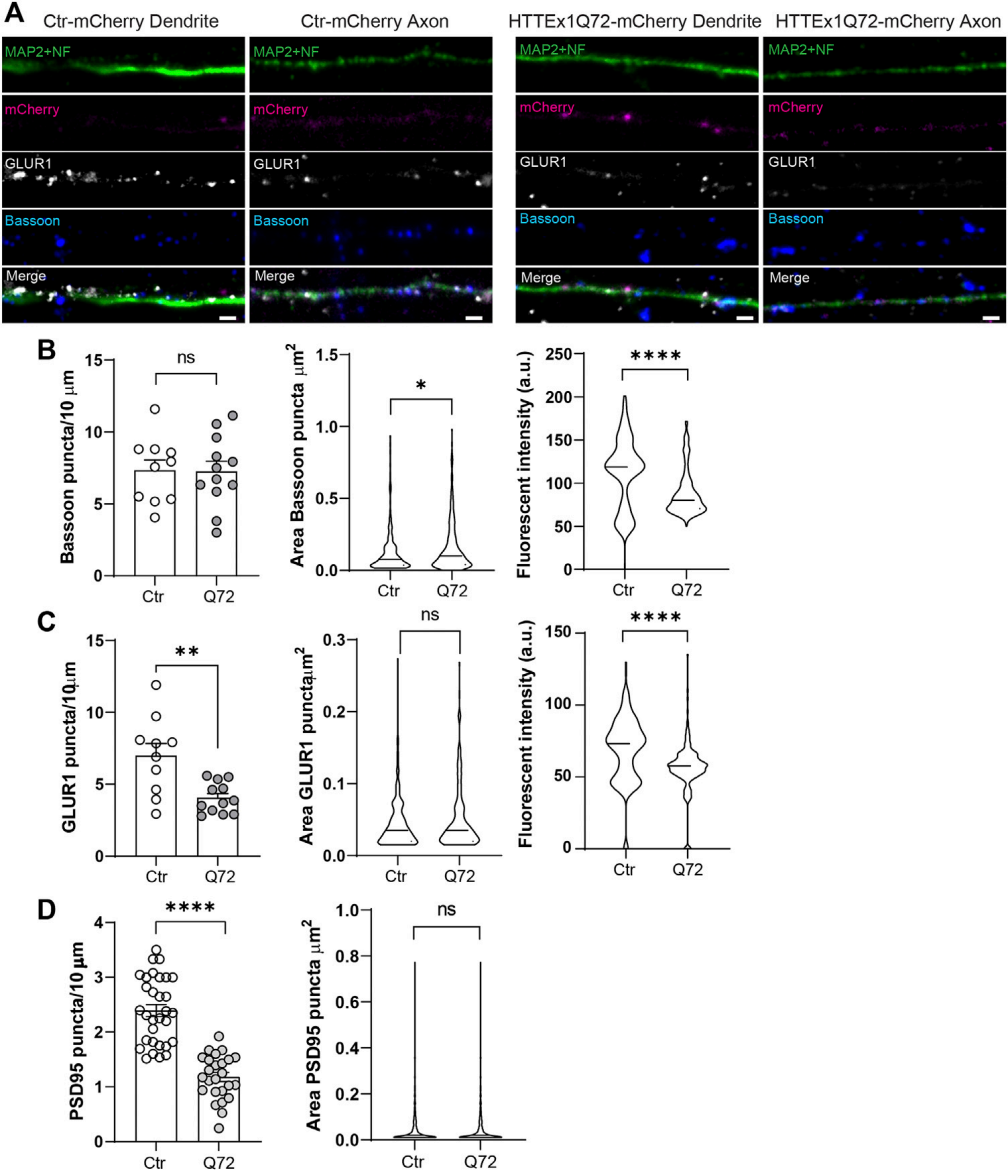
Synaptic protein distribution is affected in HTTEx1Q72-mCherry neurons. **(A)** Indirect immunofluorescence representative images of neurites from Ctr-mCherry and HTTEx1Q72-mCherry neurons on DIV 30 visualizing MAP2 and NF together, mCherry, and the synaptic markers Bassoon and GLUR1. **(B)** Quantification of the number of puncta, puncta area, and fluorescence intensity for presynaptic marker Bassoon in Ctr-mCherry and HTTEx1Q72-mCherry neurons on DIV 30 (*n* = 10–15 cells from three independent cultures). **(C)** Quantification of the number of puncta, puncta area, and fluorescence intensity for postsynaptic marker GLUR1 in Ctr-mCherry and HTTEx1Q72-mCherry neurons on DIV 30 (*n* = 10–12 cells from three independent cultures). **(D)** Quantification of the number of puncta and puncta area for postsynaptic marker PSD95 in Ctr-mCherry and HTTEx1Q72-mCherry neurons on DIV 30 (*n* = 25–30 cells from 3 independent cultures). Scale bar = 5 µm. All averaged data are shown as mean ± s.e.m. (*p < 0.05, **p < 0.005, and ***p < 0.0001, ns = non-significant, Student’s t-test (Abbr: MAP2: microtubule-associated protein 2, NF: neurofilament, GLUR1: Glutamate receptor 1 subunit, PSD95: Postsynaptic density protein 95)

We next evaluated the distribution of Bassoon, GLUR1, and the postsynaptic density protein PSD95 along the neurites on DIV 30 (Figure 2A). To label all the neurites, we used a mix of antibodies for microtubule-associated protein 2 (MAP2, dendrite marker) and neurofilament (NF, mainly axonal marker). On DIV 30, HTTEx1Q72-mCherry neurons showed a synaptic structural disruption with a significant increase in the area, a decrease in the fluorescence intensity, and no significant difference in the number of Bassoon puncta compared to Ctr-mCherry (Figure 2B). On the postsynaptic side, we observed a decrease in the number of GLUR1 and PSD95 puncta in HTTEx1Q72-mCherry compared to Ctr-mCherry neurons, with no differences in the puncta area of both proteins, but a significant decrease in GLUR1 intensity in HTTEx1Q72-mCherry (Figures 2C,D). These results suggest that the initial observation of reduced neuronal maturation in HTTEx1Q72-mCherry neurons on DIV 7 is also reflected in a more mature system (DIV 30). Thus, the distribution and expression of synaptic components are affected in mHTTEx1Q72-mCherry carrying neurons.

**FIGURE 3:**
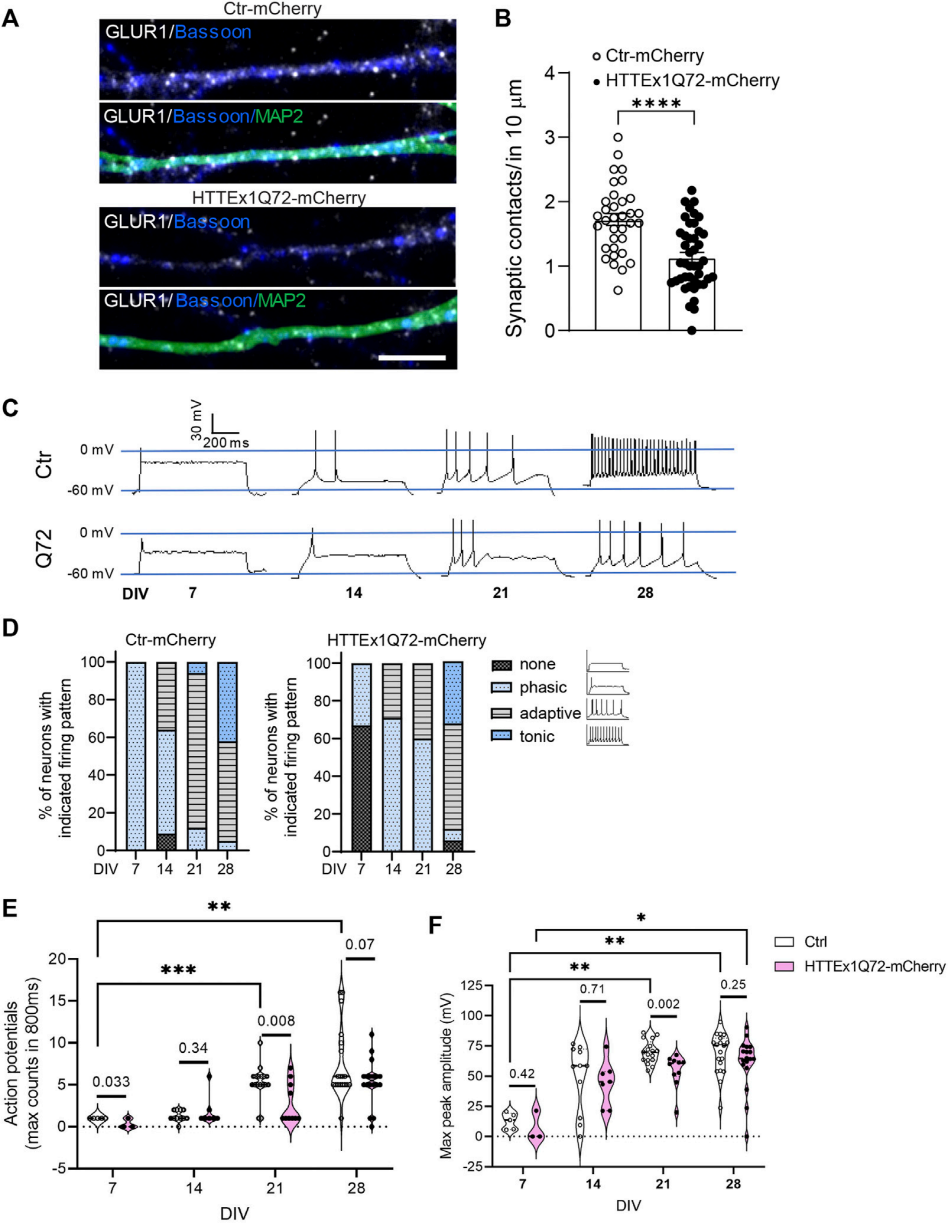
Neuronal maturation is delayed in HTTEx1Q72-mCherry neurons. **(A)** Indirect immunofluorescence representative images of dendrites from Ctr-mCherry and HTTEx1Q72-mCherry neurons on DIV 30 visualizing MAP2 and the synaptic markers Bassoon and GLUR1. **(B)** Quantification of the number of synaptic contacts, defined as close opposition between GLUR1 and Bassoon in 10 µm of dendrites (*n* = 30–40 cells from three independent cultures; *p* < 0.0001****unpaired Student's t-test). **(C)** Representative current-induced firing pattern of individual Ctr-mCherry and HTTEx1Q72-mCherry neurons at different weeks of culture. **(D)** Graphs display the percentage of neurons with indicated current-induced firing patterns recorded on DIV 7–28. **(E,F)** Quantification of the maximum number of action potentials counted in 800 ms **(E)** and quantification of the maximum peak amplitude **(F)** measured on DIV 7–28 (*n* = for DIV 7: 3–5, for DIV 14–28: 10–20; *p* < 0.03*, *p* < 0.002**, and *p* < 0.0002***, two-way ANOVA mixed-effect model; exact *p*-values: unpaired Student's t-test. Scale bar = 5 µm. All averaged data are shown as mean ± s.e.m.

Finally, we decided to evaluate whether these alterations in synaptic proteins in HTTEx1Q72-mCherry neurons correlated with changes in synaptic number and neuronal function. We quantified the synaptic contacts (opposition of Bassoon and GLUR1 staining) along neurites. In HTTEx1Q72-mCherry neurons, there was a significant decrease in the number of synaptic contacts compared to Ctr-mCherry neurons (Figures 3A,B). At the functional level, patch-clamp recordings from Ctr-mCherry neurons at increasing culture time points revealed the functional maturation of an intrinsic, current-induced action potential firing pattern from phasic at 1 week to mostly mixed phasic/adaptive at 2 weeks and developed to mostly adaptive with some neurons displaying a tonic pattern at 3 weeks. At 4 weeks, a mixed adaptive/tonic pattern had developed (Figures 3C,D left panel). HTTEx1Q72-mCherry showed a mixed none/phasic at 1 week, at 2 and 3 weeks of culture, the pattern was similar, displaying a mixed phasic/adaptive. At 4 weeks, the pattern then resembled that of Ctr-mCherry neurons, with the appearance of tonic firing (Figure 3D, right panel). The maximum action potential (AP) counts were significantly higher on DIV 21 and 28 compared to DIV 7 in Ctr-mCherry neurons, whereas HTTEx1Q72-mCherry neurons showed a tendency for increased AP number with development, but it was not significant (Figure 3E). The maximum peak amplitude also increased with development and was significantly larger on DIV 21 and 28 than on DIV 7 in Ctr-mCherry neurons. In HTTEx1Q72-mCherry neurons, the maximum peak amplitude was only significantly larger on DIV 28 compared to DIV 7 (Figure 3F). Both the action potential number and maximum peak amplitude were significantly smaller in HTTEx1Q72-mCherry than in Ctr-mCherry on DIV 21 (Figures 3E,F). This demonstrates a slowing down in the maturation of an intrinsic neuronal action potential firing pattern. Together, in this work, we show a valid human system that rapidly generates functional forebrain neurons, which allowed us to study neurodevelopment in a shortened time window. Furthermore, our results suggest that the presence of mHTTEx1 in human neurons causes a delay in molecular, structural, and functional maturation.

## Discussion

Wild-type HTT is essential for development, as its knockout is embryonic lethal (Duyao et al., 1995; Nasir et al., 1995; Zeitlin et al., 1995). It is implicated in virtually all major facets of synaptic neurotransmission including anterograde and retrograde transport of proteins to/from terminal buttons and dendrites, neurotransmitter release, endocytic vesicle recycling, and postsynaptic receptor localization and recycling (Barnat et al., 2020; Barron et al., 2021). These processes are all affected in mature neuronal networks in HD models (Metzler et al., 2003; Milnerwood and Raymond, 2010). Our data show that mHTTEx1 expression in hiPSC-derived forebrain-like neurons interferes with neuronal maturation, reduces the number of neurites, alters the appearance of pre- and postsynaptic proteins, and causes a reduction in synaptic contacts and a delay in the development of an intrinsic neuronal action potential firing pattern. Similar findings were observed in a study by Mehta et al (2018) in HD patient hiPSC-derived cortical neurons, although in the phenotypes first appeared after a much longer culture time than in our study (60–80 versus 21 days in culture) (Mehta et al., 2018). The NGN2 hiPSC-derived neuronal system expressing a tag-fused version of only mHTTEx1 used in our study is thus a valid system to study HD-related questions concerning early neuronal development, with the advantage that the specific phenotypes occur within a much shorter time window.

The study by Mehta et al (2018) additionally revealed a delayed onset of spontaneous activity in the HD cortical neurons. The NGN2 hiPSC-derived neurons also generate spontaneous activity already on day 14 in culture (Nehme et al., 2018). In the present study, we did not assess the development of the spontaneous activity, but we did find dysregulation of dendritic arborization, synaptic connectivity, and potential insertion of AMPA receptors in the postsynaptic membrane, all phenotypes regulated by spontaneous activity during early neuronal development (Moody and Bosma, 2005).

Furthermore, our data suggest that the presence of mHTTEx1 affects differentially pre- and post-synaptic assembly: as early as 7 days in culture, we could observe that the clusters of the presynaptic scaffold protein Bassoon seemed to be larger whereas the number of the postsynaptic AMPA receptor subunit GLUR1 was significantly smaller in HTTEx1Q72-mCherry than in Ctr-mCherry neurons (Figures 1E–G). At 30 days in culture, the Bassoon clusters were still larger, and additionally, the fluorescent intensity was significantly decreased in HTTEx1Q72-mCherry compared to Ctr-mCherry (Figure 2C). Bassoon binds indirectly to mHTT and in the R6/1 mouse model of HD, it has been found that Bassoon is sequestered into the mHTT aggregates (Ackermann et al., 2019; Huang et al., 2020). Furthermore, in HD neuronal cultures and mouse models, the *HTT* mutation results in decreased levels of both Bassoon mRNA and protein (Huang et al., 2020). The reduced concentration of Bassoon in presynaptic buttons found in our study might be explained by one or both of these mechanisms.

Finally, we observed a decrease in the puncta of the AMPA receptor subunit GLUR1 and PSD95 along the dendrites (Figure 2C). It has been shown that AMPAR surface diffusion is disturbed in various rodent models of HD (Zhang et al., 2018). In addition, wild-type HTT, through its association with HIP14 and PSD-95, regulates synaptic receptor stabilization at the postsynaptic density in neurons, and wild-type HTT loss may reduce the number of trapping slots for AMPA receptor insertion during long-term potentiation, by reducing the clustering of PSD95 at postsynaptic sites (Opazo et al., 2012; Parsons et al., 2014; Barron et al., 2021). This evidence, together with our data, suggests that the observed decrease in AMPAR and PSD95 could translate into impaired synaptic plasticity, a common mechanism underlying cognitive and psychiatric disturbance observed early in HD patients.

The reduced intensity of Bassoon at the presynaptic sites and the decreased intrinsic capacity of action potential firing might result in reduced neurotransmitter release (Sudhof, 2012; Huang et al., 2020), together with the observed reduction in GLUR1 at the postsynaptic side, is likely to result in impaired neuronal communication. Synapse formation in the central nervous system is regulated by pre- and postsynaptic neuronal activity, and thus, the reduced synapse number that we observed in neurons expressing mHTTEx1 could partially result from the aforementioned phenotypes induced by mHTTEx1 (Goda and Davis, 2003).

Altogether, our data suggest that the presence of mHTTEx1, during the time of synapse assembly, might alter neuronal properties crucial for the correct assembly and function of a neuronal network. Inappropriately assembled neuronal networks might represent a structure with overall weakened connections, increasing its vulnerability to degeneration. In this context, it is interesting to mention that studies, which visualized axonal connectivity tracts by diffusion magnetic resonance imaging (dMRI), revealed already at preclinical stages of HD a significant loss of white and gray matter connectivity both in the HD mouse line R6/1 and in HD patients (Poudel et al., 2014; Gatto, 2021; Gatto et al., 2021).

To better understand the impact HD mutation has on early synaptic development in HD and the long-term consequences thereof for brain function, it is essential to extend this work to HD model systems that more closely resemble the physiological *in vivo* environment of the disease. A more complex cellular environment is important, in particular, including astrocytes. Astrocytes play an important role in synapse maturation and have been shown to be affected already at preclinical stages in HD R6/1 model mice (Gatto et al., 2021). Furthermore, the HD mutation results in both a gain-of-function due to the presence of mHTT and a loss-of-function due to reduced levels of wild-type HTT. The loss of wild-type HTT has been shown to also alter synapse development; thus, the true outcome of the disease is likely determined by the naturally occurring balance between mutant and wild-type HTT (McKinstry et al., 2014). Developmental alterations can thus be most precisely assessed in genetically correct HD hiPSC-derived brain organoids, but revealing the long-term consequences of these potential developmental alterations for adult brain function will depend on *in vivo* studies using those transgenic HD mouse models which allow an earlier onset and faster progression of pathology.

## Data Availability

The original contributions presented in the study are included in the article/Supplementary Material; further inquiries can be directed to the corresponding author.
