# Supplementary figures and images for: Synaptic and functional alterations in the development of mutant huntingtin expressing hiPSC‐derived neurons
Source: Front Mol Biosci. 2022 Jul 19;9:916019. doi: 10.3389/fmolb.2022.916019 (PMC9343803; doi:10.3389/fmolb.2022.916019)

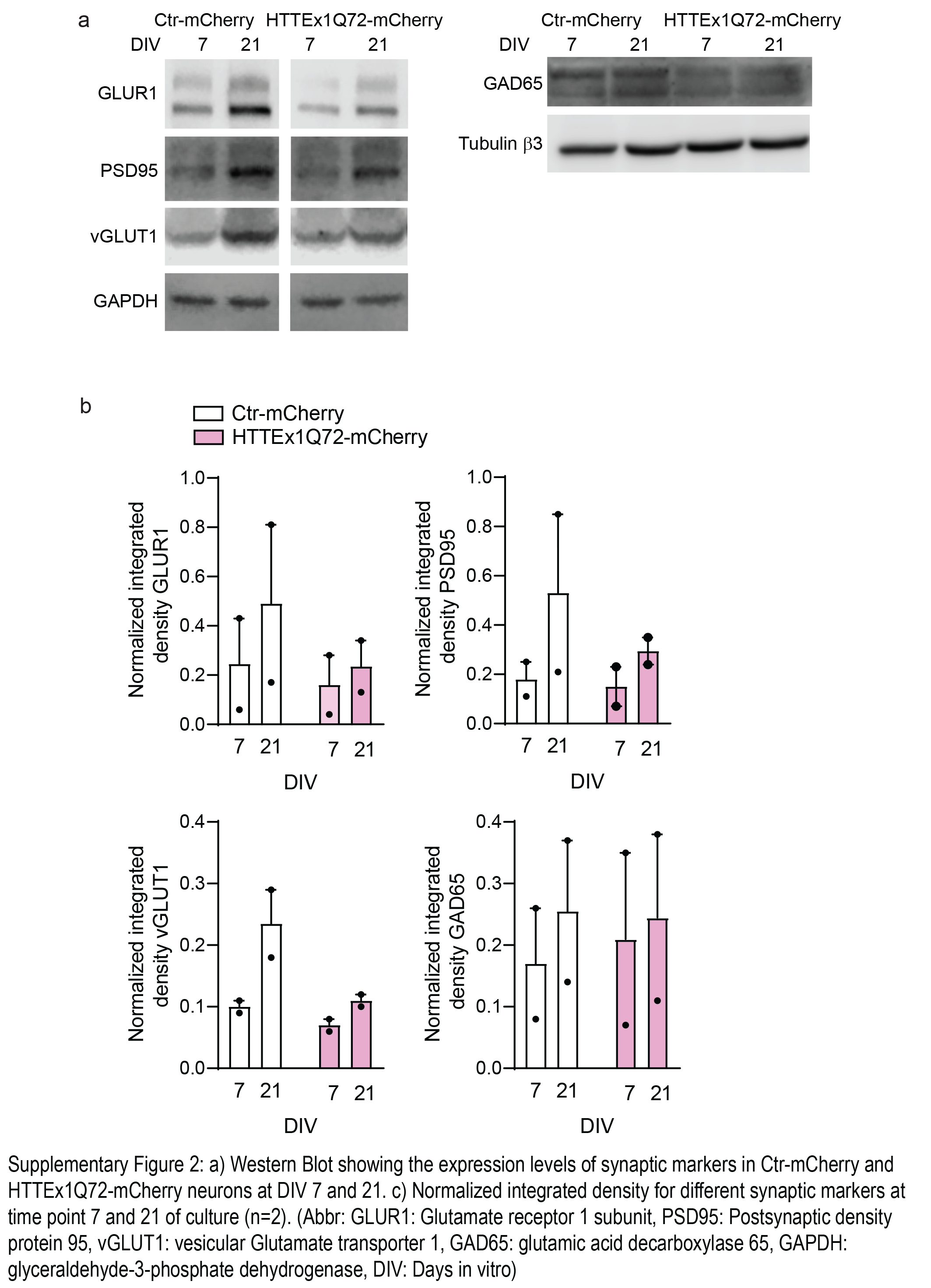

Supplement: Supplementary file 1 [file Image2.TIF]

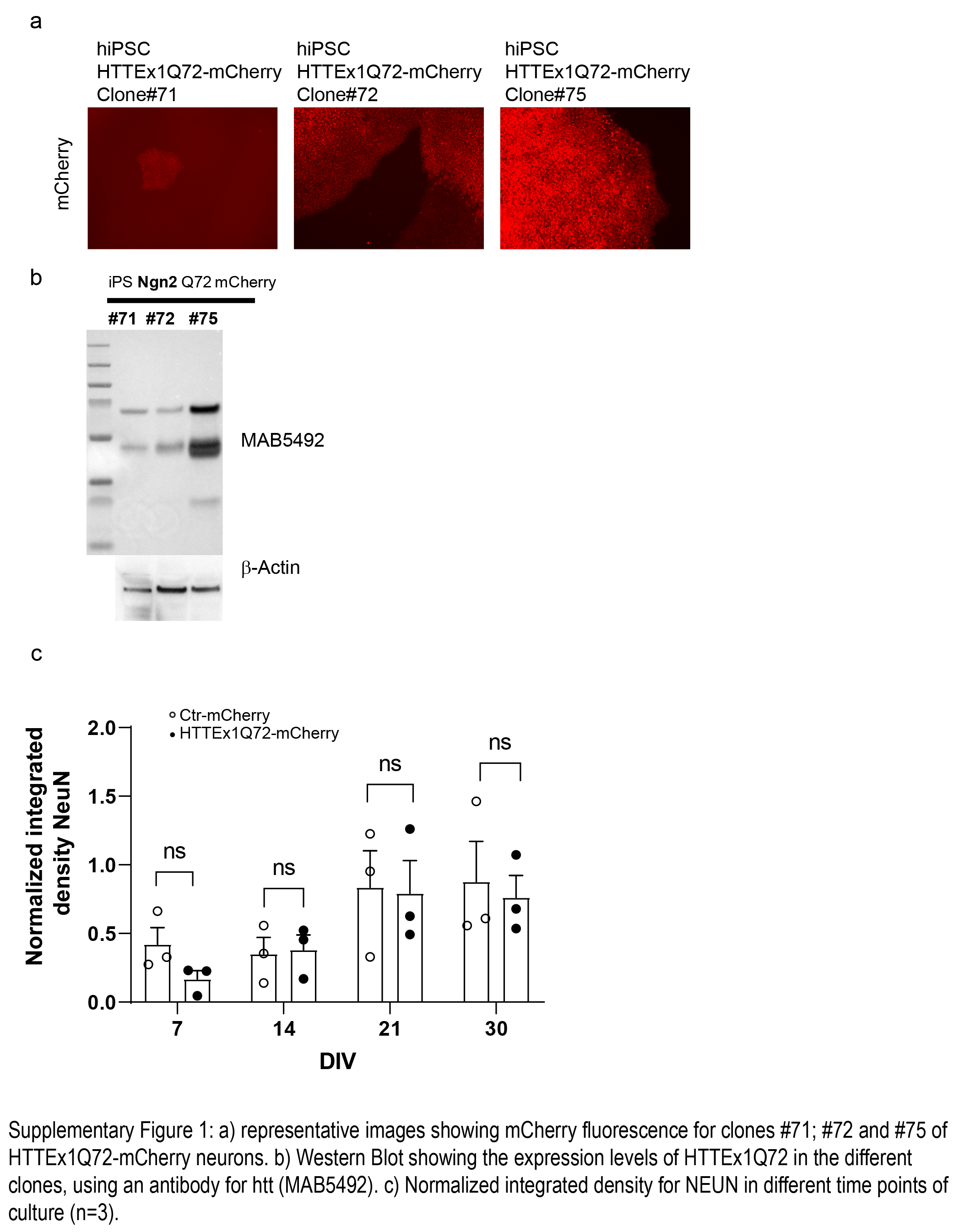

Supplement: Supplementary file 2 [file Image1.TIF]
